# State Legislation Respecting Medical Practice

**Published:** 1844-10

**Authors:** 


### ILLINOIS

### MEDICAL & SURGICAL JOURNAL.

VOL. I. OCTOBER, 1844. NO. 7

### STATE LEGISLATION RESPECTING MEDICAL PRACTICE.

As the period for the session of the Legislature approaches, we perceive a disposition on the part of many members of the Profession to agitate the subject of "medical legislation," and it becomes a question of serious importance to all, whether any, and if any, what legislative action should be asked for.

There are at present no special legislative enactments relating to the practice of medicine in the State of Illinois. Every one is entitled to assume to himself the title of M. D., to prescribe any or all the substances in the three kingdoms of Nature, to any who call upon him for advice, and to collect his bills according to the provisions of the common law. There was formerly a law "regulating the practice of physic and surgery," but this was repealed. The ground is then clear, and it remains but to be ascertained what is desired by the profession, and what will be useful to the public. Various communications, verbal, written or printed, have been made to us, or have fallen under our notice, from Physicians of great respectability, having reference to the subject, and suggesting different modes of proceeding.

1st. It is proposed by some, that petitions be presented for the passage of a law organizing district medical societies, giving them power to decide upon the qualifications of physicians coming to practice within their limits, and prohibiting all persons not licensed by such societies, from practicing medicine under heavy penalties.

2d. Others propose the organization of societies, but no penalties upon unlicensed practitioners, prohibiting them only from exacting pay for their services in the courts of justice.

3d. There are others still who wish simply that the graduates and licentiates of certain districts be incorporated into medical societies, having only the powers of scientific associations, and for their objects only the improvement of their members.

In reference to the first of these plans, the prohibition of unlicensed persons by penalties from practicing, abundant experience has, we think, shown that in the different States of this Union, such laws are entirely ineffectual. Look at the examples of New York, Ohio, Delaware, and other States where these laws have existed, and where they have only served to keep the medical societies arrayed against quacks; and where these latter, by raising the cry of persecution, have enlisted popular sympathy, and by applications to the different legislatures, have obtained a repeal of such statutes. These contests have been degrading, the results mortifying, and even a triumph would have been barren. Let us be warned by their example, not to enter upon a course from which there is neither honor nor advantage to be derived. Let no one suppose that we underrate the evils resulting from such persons being allowed to practice. On the contrary, numerous are the instances within our own knowledge, in which limbs and life have been sacrificed to their ignorance, rashness or inefficiency. Many such examples of suffering and misery from their treatment, are fresh in our recollection. We believe, too, what some of our friends doubt, that such laws are just, and no infringement of private rights; but we are convinced that in operation, they neither protect the public nor benefit the profession; but only serve to raise into undeserved notice those against whom they are directed. Nor is the expedient of excluding them from courts of justice in the collection of their bills, more effectual, since it actually gives them a pretence for expecting payment in advance, or puts their demands in the light of debts of honor.

We are opposed to all restrictive laws in regard to the practice of medicine. We have not room in this Journal, to do more than glance at the reasons that have led to the formation of this opinion. But it is a subject on which we have long reflected—we have observed the course of medical legislation—we have consulted some of the most experienced and judicious members of the profession, and there is, we think, at present, no doubt remaining, as to the proper course to be pursued,—it is to ask for no restrictive legislation in regard to medical practice.

If it should be thought desirable to have medical societies incorporated, we have no objection to this course, as it has reference to the profession and its improvement; and will give physicians the benefits of association more perfectly than can be obtained without the aid of charters. These benefits are the cultivation of friendly feeling—the keeping alive of interest in medical improvements—the establishing of libraries, &c. We exclude the right of judging of the qualifications of physicians, except their own members. There is an argument in favor of these societies, which is: that one of the greatest evils with which the profession has to contend, is not the intrusion of irregular practitioners, as Thompsonians, &c.; but a great number of the professed regular practitioners, who, however, are without diplomas, having only been engaged in medical studies for a few months, and attended a single course of lectures. They assume the title of M. D., and the public suppose them possessed of diplomas. If there were organized Medical Societies in existence from which such persons were excluded, it would act as a powerful check on their pretensions.

We will, at some future time, have something to say as to the best means of improving the character of the profession, which is not to be done by legislative enactments, but by improving the Medical Schools, discouraging unqualified persons from entering upon the study, founding associations for medical improvement, &c. At present we shall cut short these remarks, in order to give place to part of the report of a committee of the "Albany County Medical Society," and we present it not only from the soundness of its views, and the high respectability of the Society by which it was adopted, but also because it has received the approbation of the medical public in every part of the Union.

County and State Medical Societies were incorporated, and the terms of admission into the County Societies were prescribed by law. Members of the County Societies were the only licensed practitioners of physic and surgery.

All unlicensed persons, except "botanic doctors," were prohibited from practising under penalty of $25 for each offence.—All unlicensed persons, without exception, were made incapable of enforcing, by legal process, the payment of compensation for services rendered to the sick.

By the act of May 6, 1844, all unlicensed persons are freed from the penalty for practising, and the disability of collecting pay for their services. Besides this, they are made liable to civil and criminal prosecutions for malpractice, gross ignorance and immoral conduct. Previous to the passage of this act, the law prescribed the mode of becoming a licensed practitioner of medicine, and conferred on such, and on the botanic doctors, the exclusive right to practise. Since the passage of this act, the law still prescribes the mode of becoming a licensed practitioner, but gives to all persons of whatever age, or sex, or education, the right to practice, and to enforce the payment of compensation for services. Hence, although the organization of the County and State Societies is left as before, it is no longer obligatory on those who practise physic and surgery to become members of the County Societies, nor to go through the course of study and the examination requisite for admission into these societies. They have become voluntary associations, which give to their members the title of licensed practitioners, but confer on them no legal rights. Such is the operation of this act on the laws regulating medical practice.

We now proceed to the examination of the question, whether the passage of this act calls for any movement on the part of the Society. But first of all, it will be necessary to review the course of legislation in regard to medical practice, to establish the principles on which such legislation ought to be founded.

Seeing what important duties devolve upon the physician, what weighty interests are confided to his skill and integrity, subject to no control but his own conscience, legislators have always recognized the propriety and necessity of providing men to assume those duties who could offer some guarantees of capacity and honesty, and of guarding the public against imposition by the ignorant and unprincipled. Hence laws have been enacted, having in view the two-fold object of raising up and organizing a body of competent physicians, and of protecting the public against imposition.

To accomplish the former of these objects, the profession has been organized by the establishment of County Societies, so that its members may be readily recognized by each other and by the public, may exercise a general supervision over each other, and co-operate to promote the common welfare.

Provision has been made for medical education by the establishment of schools liberally endowed, in which students may, at moderate expense, be taught the science and art of medicine.—A course of study has been prescribed, through which candidates are required to pass before they can be admitted to an examination by which their qualifications are to be tested. After having accomplished this course of study and passed the examination, the student is admitted into the profession as one worthy of its honors and fitted to assume its duties.

Thus are attained the first great objects of medical legislation. A body of physicians is created, presenting certain guarantees of capacity and character, and this body is organized so that its members may be readily recognized by the public. These objects and the means by which they are attained we all unite in commending. If any person, with these means of choosing, applies for medical aid to one who can offer no guarantees of proper qualifications, he is guilty of a gross imprudence; but it is at his own risk, and he has to suffer in his own person all the consequences. The law has protected him against imposition, but not against a foolish choice.

It might be supposed, that when men have the choice before them of educated physicians, presenting evidences of their qualifications, and of others whose main titles seem to be their ignorance and impudence, they would not hesitate to have recourse to the former. But sad experience shows that this is far from being true. We find that men who conduct all their other affairs with prudence and discretion, are willing to abandon a medical attendant of tried skill and character, for any juggling mountebank whose pretentions would only excite a smile, were it not for the deplorable results to which they give rise. Struck with this sad spectacle of human credulity and folly in cases in which such important interests are involved, legislators have thought that it was not sufficient to provide educated physicians, and to give the public the means of recogizing them, but have passed laws prohibiting all but regular physicians from practising. These laws are founded on the presumption, that it would be so absurd to have recourse for medical aid to an ignorant person, when it is possible to procure the services of an educated physician, that those, who might be tempted to do so, must be treated as incompetent to manage their own concerns. To prevent them, therefore, from indulging in such folly, all irregular practice is prohibited under certain penalties.

The laws for educating and organizing a body of physicians were intended to give men the means of acting prudently; the prohibitory laws were intended to compel men to act prudently. So long as public sentiment accords with this view of the legislator, the operation of these prohibitory laws is salutary; while only a very few silly persons prefer to have recourse to men out of the profession for relief, it seems proper to protect them against their own bad judgment, just as minors and imbecile persons are not allowed to make contracts by which they might be swindled by knaves. But unfortunately a large portion of the public think that education and science are not necessary to qualify men for medical practice. Numerous sects have sprung up, pretending to cure diseases by various processes, more or less ridiculous, but all agreeing in this one point, that it is not necessary to pass through the regular course of studies required by law, but that there is a royal road to medical practice which renders such drudgery useless. These sects, absurd as their doctrines may be, have succeeded in gaining followers among the public, and the effect of these restrictive laws, if enforced, must be to prevent all their followers from procuring the kind of medical aid which they prefer. Besides, it must be remarked that those who are thus placed under the legislative tutelage, are not exclusive the ignorant or imbecile, but that they number in their ranks many persons of education and sagacity, who manage all their other affairs with sufficient acuteness and discernment. However absurd the opinions and conduct of these men may appear to us, we have not, for that reason, the right to impose on them our ideas of wisdom. If, for example, a full-grown man who is capable of managing his own business, chooses to call in, to reduce a dislocation, a natural bone-setter who avows that he has never seen a skeleton, in preference to a surgeon who has devoted himself to the study of such accidents, we may deplore his folly, and endeavor to persuade him to act more prudently; but we ought not to use compulsion either directly or indirectly. If his conduct is foolish, he alone suffers from it, and as we are not responsible for his folly, we have no right to prevent him from indulging in it.

On this point we have the misfortune to differ with some for whose opinions we have great respect, and we wish to be well understood. None can be more deeply impressed than we are with the immense amount of mischief inflicted on community by irregular practitioners of medicine. We feel indignant at the base deception they daily practise under our eyes, and we pity their dupes. We all alike agree in deploring the evil, but there is some difference as to the remedy. The experiment of the past satisfies us that legislative wisdom never can restrain individual folly; that all that legislation can do in such matters is to give to all the means of knowing the character of those to whom they may apply, and thus enable them to act with a full knowledge of the circumstances, and leave the rest to man's own wisdom and prudence. We are accustomed to apply this principle to other cases of a like nature. Absurd and mischievous religious principles sometimes spring up. We are pained to see men led away by vile superstitions, or fall victims to the arts of designing leaders, yet we do not attempt to put down such systems by law, because we do not think it right to impose our religious views upon others, and because we know that any such attempt would only serve to confirm them in error. So, too, in matters of ordinary business, the law protects men against imposition so far that if one, in making a bargain, is deceived by false representations, the law would give him redress; but if, with a full knowledge of the facts, one enters into a foolish bargain, he must abide by the consequences. There is no reason why this principle should not be applied to medical practice.

But even admitting that these restrictive laws are founded on principles of sound policy and justice, there is still one objection which is unanswerable. It is entirely impossible in this country to enforce them. For many years they have been in existence, and yet men have practised under our eyes openly and avowedly in violation of them, and in no one instance has the penalty been enforced. As to the disability of recovering payment for their services by legal process, it has had quite as little influence, for we think it is altogether probable that botanic doctors, and homœopathists and other quacks, have been quite as well paid as the regular practitioners.

The practical operation of these laws was rather favorable to the class of irregular practitioners. The penalty they imposed was never regarded, the disability of collecting debts afforded a pretext for demanding payment in advance, and gave to their demands the character of debts of honor. Besides this, they put it in the power of quacks to raise a cry of persecution and represent the profession as greedy monopolists, and thus excite some feeling in their favor among weak and credulous people. A clamor for the repeal of those laws was kept up for the purpose of advertising the system rather than obtaining any rights about which they really cared, and since the repeal has been obtained they will have to devise some new plan to wriggle themselves into notice.

It will be remarked, that in all our reasoning on this subject of these restrictive laws, we have considered them as designed for the good of the public and not of the profession. This is undoubtedly the only ground on which they can be defended. The object of those who enacted them, was to protect the people against the ignorance and rapacity of quacks, and not to protect the profession in a monopoly of practice, to be enjoyed for the benefit of its members. If, in the repeal of these laws, a wrong was committed, the public and not the profession must be considered the injured party. It behooves us neither to claim as a right nor to ask as a favor any exclusive privilege, which is opposed to, or which is not directly conducive to, the public good. If these restrictive laws are not called for from considerations of public safety, then there should be no opposition on our part to that repeal. It is certain, that no class of community are so little liable to be injured by quacks as physicians who know how to avoid them.

This point has been lost sight of in the discussions on the subject in the legislature and elsewhere, and we are anxious to bring it clearly in view, because it does not comport with the dignity of our profession to appear to be engaged in a selfish contest for privilege with the different bodies of quacks which infest the community. As the natural guardians of the public interests in such matters, it is incumbent on us to admonish the legislature, if we think they are acting ignorantly or rashly, but we must be careful to have it understood that in so doing we are not defending our privileges against the rest of the public, but that we are defending the public against their own rashness and folly.

To resume. We consider that the great end of legislation in medical practice should be to provide a body of competent physicians, and to give the public the means of recognizing them, leaving to the prudence of individuals to choose discreetly; and that all attempts to coerce people to discretion are wrong in principle and unsuccessful in practice.

We are now prepared to examine the question, whether under the circumstances any action of the Society is called for?

We have expressed our views as regards the restrictive laws. Whatever difference of opinion may exist as regards the general policy of such laws, there is one point on which all must agree. It is utterly impossible to enforce them so long as they are not in accordance with public sentiment. We would, therefore, be exceedingly sorry to see the profession again entering into a contest with Thomsonians and other persons of that class, for the sake of restoring a law which we know before hand cannot be executed, and which serves as a pretext for quacks of all kinds to raise the cry of persecution, and to represent the profession as made up of selfish monopolists—a contest in which defeat would be mortifying, and success would bring no real advantage.

We are aware that much feeling has been excited in the profession by the repeal of the laws, but this is owing rather to the manner in which it was effected and the ground on which it was urged, than to the act itself. Although it was sustained by some for proper reasons, yet a few senseless demagogues in the legislature, fit organs of the quacks, whose cause they espoused, did not fail to seize that occasion to revile the whole body of physicians, and to represent them as engaged in a struggle to maintain a monopoly of practice in their own hands. The profession was thus placed in a false position; it appeared to be fighting for its privileges against the quacks; the interest of the public in the contest was kept out of view, and the result was hailed as a triumph of quackery over the medical profession. We hope that in future they will be allowed to enjoy their triumph without any interference on our part. We should be sorry to become engaged in a contest with ignoble adversaries for the benefit of a public which will always look upon our mediation with suspicion. Let the knaves and the dupes in future settle their accounts among themselves.

As regards the laws regulating medical education and the organization of the profession, we do not know of any modification which would be desirable. The State and County Societies have all the powers necessary to enable the profession to act with unity and efficiency. What is still wanting here, depends not on the legislature, but on ourselves. We ought to endeavor to infuse more spirit into our County Societies, to have more frequent meetings, and to promote cordiality of feeling among its members. The rules of medical ethics should be scrupulously observed, and any violation of them promptly noticed by the Society.

In the law of last winter, an amendment was offered requiring unlicensed practitioners to express their true character by having the word "unlicensed" on their signs. This amendment, to which no sound objection could be made, since it could only serve to inform the public of the true character of those who offered their services, and which, if one half was true of what was said in debate respecting the superiority of Indian doctors, homœopaths and steam doctors over the regular profession, would have conferred a real advantage on the unlicensed practitioners, was rejected. Although we think the amendment a good one, yet we should be sorry to go again before the Legislature to ask for its passage, and we think the same end might be attained if every County Society would publish in the newspapers semi-annual or quarterly lists of their members.

Now that all restrictions on practice are removed, it will be practicable to raise the standard of admission into the County Societies without exciting any well-founded opposition. These societies are now voluntary associations, into which those who find the requirements too high need not enter. A well-matured plan, which would increase the amount of requisitions without putting it at a point unattainable at the present time, would no doubt be favorably received by the profession.

We would then say, in conclusion, we have laws enough, and good laws. Quackery must be suppressed not by legislation, but by enlightening the public as to its dangers. The dignity and respectability of our profession is to be promoted not by asking for legal privileges, but by an increase of individual zeal and a more cordial coöperation. It is a great error to suppose that the repeal of the restrictive laws puts the physician on a level with the quack and takes away the barrier which separated them. The barrier which effectually separates the two classes is formed by the higher attainments and honorable deportment of the members of the former, and this is the barrier which it depends on us to make higher and stronger. It is one which quackery will not surmount, and which legislative enactments cannot break down.

In accordance with these views, the committee offer the following resolution:—

*Resolved*, That in the opinion of this Society, it would not be conducive to the interest or respectability of the medical profession, at the present time, to apply to the Legislature for any alteration in the charters of the State or County Medical Societies; or any legislation on medical subjects whatever.

THOMAS HUN,
JOEL A. WING.
MASON F. COGSWELL.

